# Debelalactone Prevents Hepatic Cancer via Diminishing the Inflammatory Response and Oxidative Stress on Male Wistar Rats

**DOI:** 10.3390/molecules27144499

**Published:** 2022-07-14

**Authors:** Prateek Pathak, Vikas Kumar, Habibullah Khalilullah, Maria Grishina, HariOm Singh, Amita Verma

**Affiliations:** 1 Laboratory of Computational Modeling of Drugs, Higher Medical and Biological School, South Ural State University, 454008 Chelyabinsk, Russia; patkhakp@susu.ru (P.P.); grishinama@susu.ru (M.G.); 2 Natural Product Drug Discovery Laboratory, Department of Pharmaceutical Sciences, Sam Higginbottom University of Agriculture, Technology and Sciences, Prayagraj 211007, India; vikas.kumar@shiats.edu.in or; 3 Department of Pharmaceutical Chemistry and Pharmacognosy, Unaizah College of Pharmacy, Qassim University, Buraydah 51911, Saudi Arabia; h.abdulaziz@qu.edu.sa; 4 Department of Molecular Biology, ICMR-National Aids Research Institute, Pune 411026, India; 5 Bioorganic and Medicinal Chemistry Research Laboratory, Department of Pharmaceutical Sciences, Sam Higginbottom University of Agriculture, Technology and Sciences, Prayagraj 211007, India

**Keywords:** hepatic cancer, debelalactone, oxidative stress, *Phyllanthus debilis*

## Abstract

The current study was conducted to exemplify the effect of debelalactone on tissue protection, chronic hepatic inflammation, hepatic protection and oxidative stress induced by diethyl nitrosamine in Wistar rats. Therefore, DEN (200 mg/kg) was used for the induction the hepatocellular carcinoma (HCC) and the level of serum alpha fetoprotein was used for the estimation and confirmation of HCC. The study illustrated that debelalactone (DL) significantly downregulated the hepatic, non-hepatic parameters such as aspartate aminotransferase, alanine aminotransferase, alpha fetoprotein, NO levels, total protein, albumin, blood urea nitrogen, total bilirubin, and direct bilirubin in dose dependent manner, as well as noticeably improving the body weight, of treated animals. The macroscopically observation of DEN-induced rat liver showed the formation of informalities in liver tissue, which was reduced with treatment of DL at dose dependent manner. However, antioxidant markers and inflammatory mediators such as lipid peroxidation, catalase, superoxide dismutase, glutathione peroxidase and transferase, TNF-α, IL-1β, IL-6, and NF-kB restored up to the normal level by DL. The histopathology studies showed that the treated group of animals returned to a normal status. Collectively, it can be concluded that debelalactone mediated chemoprevention in the DEN-induced rats via an increase in the activities of endogenous enzymes and/or inhibition the precancerous cells.

## 1. Introduction

Cancer is multistep, multi-factorial disease with development, progression and initiation of carcinogenesis via an accumulation of multiple hits, involving the epigenetic and genetic alterations [1]. Hepato-cellular carcinoma (HCC) is the 5th most common cancer among the other types of cancers worldwide [2]. In all type of cancers, HCC represents more than 4% cases and every year 440,000 new cases are globally detected [3]. At this time, transplantation and surgical resection are only possible treatment against HCC, which are applied to only in few patients with early detection of tumors. Apart from these, other alternative methods of treatment are also available which include chemotherapy, radiotherapy, and surgery. Therefore, the methodology of the treatment depends upon cancer types and stages [3,4,5]. The treatment of liver cancer is predominantly challenging due to various factor such as patient medical history, liver (parenchymal), and tumor (size, shape, number, vascular movement, and stage) variables. The earlier discussed treatment having higher toxicity and developing drug resistance, which not only effect tumor cells but also effect normal cells, can also lead to various side effects such as thrombocytopenia, nausea, diarrhea, constipation, and alopecia [4,6,7]. Chemoprevention consists of the management of cancer by targeting various signaling pathways [3,4]. Cancer chemoprevention is an intervention of synthetic and herbal products, which may be inhibits irregular growth of cancer cells [5]. Studies illustrated that hepatocarcinoma increases the level of free radicals due to the burst release of cellular injury and oxidative stress [8,9]. It is well known that oxidative stress plays an important role during hepatocarcinogenesis. The level of reactive oxygen species (ROS) increases during oxidative stress, which play a causative role to induction of DNA injury and tissue damage or both. It was also observed that the risk of morbidity and mortality from COVID-19 are advanced in the cancer patients than normal population [10]. Therefore, widespread global changes in the patterns of prescribing chemotherapy and anticancer treatment are introduced [11]. The current approach of cancer prevention of using plant-based drugs and their isolate compound, dietary product or synthetic compounds serve as prevent or suppress the progression of malignancy, has become popular with increasing cases of cancer globally. [12] Some of epidemiology studies shows that the regular consumption of vegetables and food can activate the anti-carcinogens and decrease the harmful substances which are increasing the risk of chronic diseases, especially cancer. [9,13] The regular intake of herbal foods and vegetables can increase the amount of beneficial nutrient viz., unsaturated fatty acids, flavonoids, selenium, terpenes, polyphenolic terpenoids etc.

**Figure 1:**
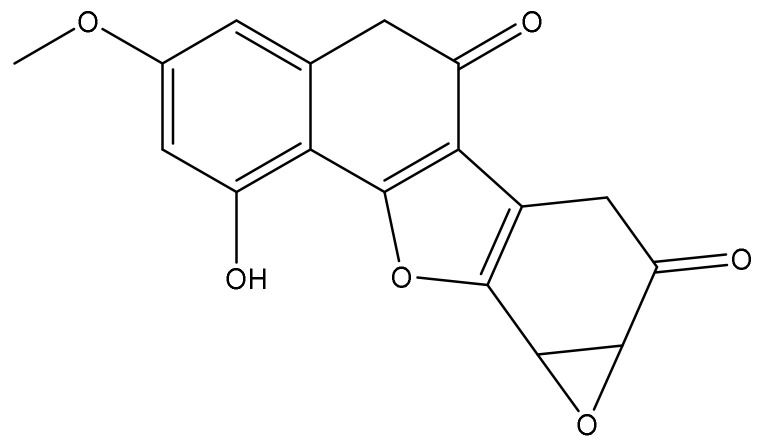
Structure of debelalactone.

*Phyllanthus debilis* belongs to Euphorbiaceae family and well spread in Western Ghat of India, Indonesia, and Sri Lanka. Various studies [14,15,16] have reported that the plant consists of a variety of pharmacological activities such as anti-inflammatory, analgesic, immunomodulatory, and hepatoprotective. In previous study, [17] our team isolated a novel oxirano-furanocoumarin debelalactone (DL) (Figure 1) from the whole plant of *Phyllanthus debilis* illustrated that DL is significantly active as a hepatoprotective. Thus, in the present study, we evaluated anti-hepatic cancer activity of DL against DEN-induced Swiss albino Wistar rat model.

## 2. Results

### 2.1. Anticancer Activity

#### 2.1.1. Effect of DL on Body and Hepatic Weight

Total body weight loss and increment of liver weight is a major complication of liver cancer, caused by a deprivation or loss of structural proteins. Therefore, we also assessed DL effects on body and hepatic weight of the experimental animals. DEN-induced experimental rats displayed a slight declined the weight as compared to the treated groups. On the other hand, DL treated groups (at 2.5 mg/kg, 5 mg/kg and 10 mg/kg) maintained the body weight till end of the study. Contrarily, the weight of the liver tissue was significantly (*p* < 0.001) augmented in DEN control group as compared to normal group. DL showed the increased the liver/body weight ratio as compared to normal control. In comparison with DEN control group rats, DL showed the reduced liver and relative body weight at dose dependent manner. The results are presented in Table 1.

#### 2.1.2. Effect of DL on Enzymatic Liver Parameters

This section of the experiment aimed to study the effect of DL on the non-hepatic serum enzyme markers in plasma and liver tissue. The literature has already proved that non-hepatic markers have vital protagonist against cancer. Therefore, in the case of hepatic cancer, the measurement of these markers significantly deregulated and accepted [18] for evaluating drug effectiveness. The results showed significant changes in AST, ALT, ALP and NO levels (Table 2). These results clearly show that the level of AST, ALT, ALP and NO were significantly increased in DEN group. However, in the treated groups the levels of each marker notably decreased in a dose dependent manner.

#### 2.1.3. Effect of DL on Non-Enzymatic Liver Parameters

We aimed to evaluate the DL effect on the non-hepatic serum enzyme markers in plasma and hepatic tissue of experimental animals. Studies have illustrated that that non-hepatic markers have very vital role against cancer and there deregulation leads the development and progression of various types of cancers [18]. The non-enzymatic liver parameter of DEN-induced rats were found to significantly (*p* < 0.001) potent as compared to normal control (Table 3). DEN-induced carcinogenesis rats significantly (*p* < 0.001) decreased the levels of total protein, albumin, BUN, total bilirubin, and direct bilirubin, which indicated the destitute liver function and in capability to fight against carcinogenesis. On the contrary, oral administration of the DL restoring the non-enzymatic liver parameters at dose dependent manner and claim the chemo-protective effect of DL against the DEN-induced carcinogenesis.

#### 2.1.4. Effect of DL on Hematological Parameters

The hematological parameters of DEN-induced rats showed the significant (*p* < 0.001) elevation of the hematological parameters when compared to normal control and DL normal control treated with 10 mg/kg dose of DL (Table 4). However, treated with different doses (2.5 mg/kg, 5 mg/kg and 10 mg/kg) of DL altered the hematological parameters at dose dependent manner.

#### 2.1.5. Effect of DL on Endogenous Antioxidant

This study aimed to determine the effect of DL on the plasma antioxidant status of experimental rats. Previous studies already proved that endogenous antioxidants act as a critical protective system against various cancer [19,20]. In various stages of cancer, the antioxidant system is greatly deregulated and compromised. Therefore, the efficiency of the DL against hepatic cancer also examined through the measurement of endogenous antioxidant The results showed significant changes in LPO (lipid peroxidation), CAT (catalase), SOD (superoxide dismutase), GPx (glutathione peroxidase), and GST (glutathione transferase) levels (Table 5). These analyses clearly show that the level of these antioxidant enzymes in DEN-induced group was significantly changed compared to the control. However, the DL treated groups showed significant improvement in the antioxidant status in dose dependent manner. The lipid peroxidation parameter (LPO), which is broadly impacts the production of reactive oxygen species (ROS), play a crucial role in the cancer progression, and is considered critical in peroxidation of membrane lipids. For that reason, levels of LPO were also measured. It can be clearly seen that in the DEN treated animal groups, LPO is significantly increased compared to the control group; whereas DL actively participates in lowering the levels of LPO in dose dependent manner. The level of endogenous antioxidant parameters such as CAT, SOD, GPx, and GST were significantly (*p* < 0.001) lowered in DEN-induced treated rats and restored by DL at dose dependent manner. The study conducted by Karki et al. [19] customary exhibited that the free radical generation is a major cause of cell damage and enzymatic and non-enzymatic antioxidants acting as defensive mechanisms. In the present study, we also observed this trend: DL decreases the levels of free radicals and oxidative stress. Therefore, it has a supplementary advantage and can also assist as an indicator of liver cancer chemotherapy.

#### 2.1.6. Effect of DL on Proinflammatory Markers

This study aimed to determine the effect of DL on the proinflammatory cytokine and inflammatory mediators of experimental animals. The test samples demonstrated substantial changes against proinflammatory cytokine and inflammatory mediators (Table 6). The result displayed that DL significantly dropped the level of proinflammatory mediators such as TNF-α, IL-1β, IL-6, and NF-kB compared to DEN control. On the other hand, DEN-induced group of animals displayed an extended proinflammatory cytokines, which was abridged by the DL.

### 2.2. Effect of DL on the Hepatic Histopathology

**Figure 2:**
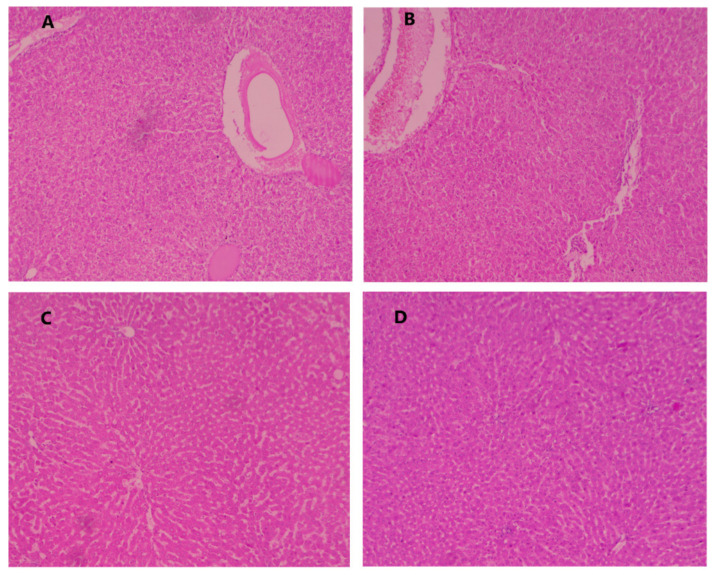
Effect of debelalactone on normal control and DENA induced tumorgenesis rats liver histopathology. (**A**): DEN Control: DENA control group rats showed the macrodroplet of fats, inflammatory blood vessels, dark and irregular shaped cytoplasm, pseudoacini and hyperchromatic nuclei. (**B**): DEN Control + debelalactone (2.5 mg/kg): DEN-induced rats treated with Debelalactone showed the deposition of macro droplet and inflammatory blood cells. (**C**): DEN Control + debelalactone (5 mg/kg): treated rats showed the less fat deposition with less inflammatory blood vessels. (**D**): DEN Control + debelalactone (10 mg/kg): treated rats showed recovery in the liver histopathology. (Original magnification 10×, DXIT 1200, Nikon, Japan).

The histopathological feature of normal control, DEN control, and DEN control treated with different doses of DL are shown in Table 7 and Figure 2. The normal control group showed typical normal architecture, polyhedral shaped hepatocytes, and nuclei of cytoplasm, granulated cytoplasm, and normal central vein. Conversely, DEN-induced group of animals exhibited cell necrosis, inflammatory blood vessels, uneven polyhedral cells with bordering wide sinusoids, small dark cytoplasm with asymmetrical shaped, basophilic, hyperchromatic nuclei, binucleated pseudoacini, uneven macro lipid droplets, eosinophilic masses in vacuolation surrounding by the cytoplasm, manifold nucleoli, enlargement of karyomegali (nuclei), hyperplasia in bile duct. The DEN-induced group also showed the propagation in portal area of hepatic stellate cells (HSCs) and causes HSCs focal proliferation. DEN-induced hepatocarcinogenesis rats treated with DL (2.5 mg/kg) showed an improved hepatocellular architecture, less inflammatory necrosis cells, altered hepatocytes, and enlarge karyomegali with presence of less micro droplet. DL treated groups (5 mg/kg) showed an improvement in hepatocellular architecture with less or more usual altered hepatocytes, less inflammatory cells.

Moreover, DEN-induced rats treated with DL (10 mg/kg) showed the compact cytoplasm, less or no presence of micro droplet with average size of mononucleated nuclei. Furthermore, the liver cells were showing the average size of karyomegali and bile duct.

### 2.3. In Silico Search for the Possible Target of DL

The search was based on a preliminary forecast of the probability of antitumor activity for 8 mechanisms, including alkylating action (Alk), antimitotics (AMi), inhibitors of topoisomerase 1 (TI-1), topoisomerase 2 (TI-2), dihydrofolate reductase (DHFR), DNA—antimetabolites (cancer DNA, or cDNA), cyclin-dependent kinase (CDK4), and epidermal growth factor receptor (EGFR). The forecast of the probability was conducted using 3D QSAR models created using CoMIn [21] models presented on www.chemosophia.com (accessed on 15 June 2021) web page were used [22]. Algorithm CoMIn superimposes structures and searches for quantitative relationships between bioactivity and the density of MERA “atomic matter” represented as:

$$\varphi_{j}=w_{ij}\alpha_{j}e^{-\beta_{j}r_{jm}^{2}}\;\;\varphi_{j}^{\prime }=-2w_{ij}\beta_{j}r_{jm}\alpha_{j}e^{-\beta_{j}r_{jm}^{2}}$$

at the junctions of a cubic grid, where $w_{ij}$ is *i*-th weight factor of atom *j* (atomic charge, volume, number of occupied atomic orbits, number of unoccupied atomic orbits, HOMO and LUMO energies as well as the products of these weight factors), $r_{jm}$ is a distance of the atom *j* from the lattice junction *m*, and $\alpha_{j}$ and $\beta_{j}$ are described in Potemkin et al., 2009 [23].

Potentials $\varphi_{j}$ and $\varphi_{j}^{\prime }$ are used as descriptors for the creation of the quantitative relationships for bioactivity (BA) description. The relationships themselves are neural network with sigmoid neurons (NNSN) and linear reaction of neural network (LRNN). Next, the calculated activity is converted into the probability of activity using the desirability function:

(1) $$p=exp\left[-exp\left(a-b\times BA\right)\right]$$

The models were created for each of the listed mechanisms of anticancer activity with crossR2 (Q2) in range 0.91–0.99. At that, each training dataset contained bioactivity data on the approved drugs. Thus, the result of superimposition can be used for restricted docking procedure (ReDock) described in Potemkin et al., 2009 [23].

For prognosis, DL has been superimposed in the generalized grid, atomic density parameters have been computed at each junction and prognosis of bioactivity has been performed using the combination of neural networks (NNSN and LRNN) and desirability function. Predicted probabilities are provided in Table 8.

Prognosis showed very promising activity of DL as DNA-antimetabolite. DNA antimetabolites are known to include approved drugs Doxorubicin and Idarubicin that are also used to treat hepatocellular carcinoma [24,25]. Therefore, docking of DL was carried out in the cDNA using a restricted docking procedure (ReDock) and QM/MM approach including MM3 molecular mechanics force field and quantum chemical method AlteQ. [26] To check the quality of the docking, the ligand of 1ims complex retrieved from Protein Data Bank was also superimposed and docked to cDNA using ReDock algorithm, the probability of bioactivity was predicted for the structure. It has been shown that the 1ims ligand possesses high probability of bioactivity.

**Figure 3:**
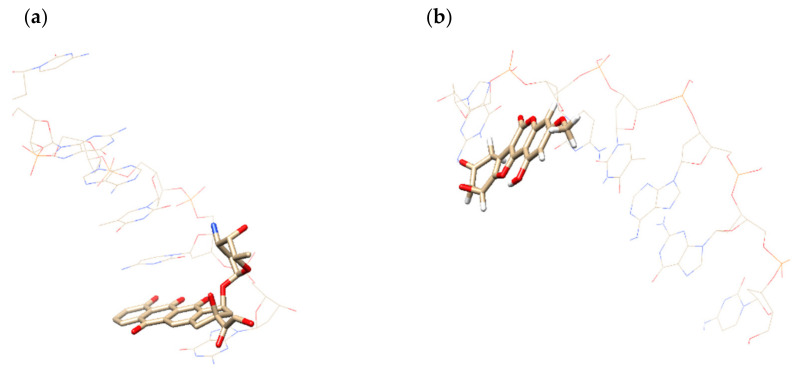
cDNA strand region—D (CGATCG) complexed to ligands: (**a**) experimental complex 1d37; (**b**) docked complex with DL.

We compared coordinates of the ligand in the experimental complex and docked complex, then we computed root-mean-square deviation (RMSD). It equals 0.80 Å for 1ims. Docked DL complex is shown in Figure 3.

DL has a planar form that is typical of DNA-antimetabolites, for example, ligands of 1d37 and 1ims complexes. In addition, the oxygen of the epoxide cycle is located below the cDNA chain, interacting with a set of water molecules, just as the oxygen atoms of ligands 1d37 and 1ims.

The complementarity assessment has been carried out using complementarity factor 1 (CF1). Squared correlation coefficients showed high complementarity of DL and cDNA comparable with experimental complexes 1d37 and 1ims. Maximum of CF1 (MaxCF1) demonstrated highly efficient interaction in the ligand-cDNA complexes; at that, there are no overlaps of inner electron shells are observed. Thus, obtained complexes don’t contradict Pauli principle. The characteristics of the complementarity assessment are given in Table 9.

Furthermore, DL demonstrated high activity against EGFR (Table 1), therefore, we can suppose that this compound can be a drug candidate against triple negative breast cancer.

### 2.4. In Silico Absorption, Distribution, Metabolic Liability Prediction, and Excretion Studies

Computational physicochemical characteristics of debelalactone were estimated to explicate important features (Table 10). Concisely, the results exhibited that DL follow of Lipinski’s rule of five [27] with optimal logP value (lower than 5), higher GI absorption rate, lower TPSA, not tendency to cross BBB.

**Figure 4:**
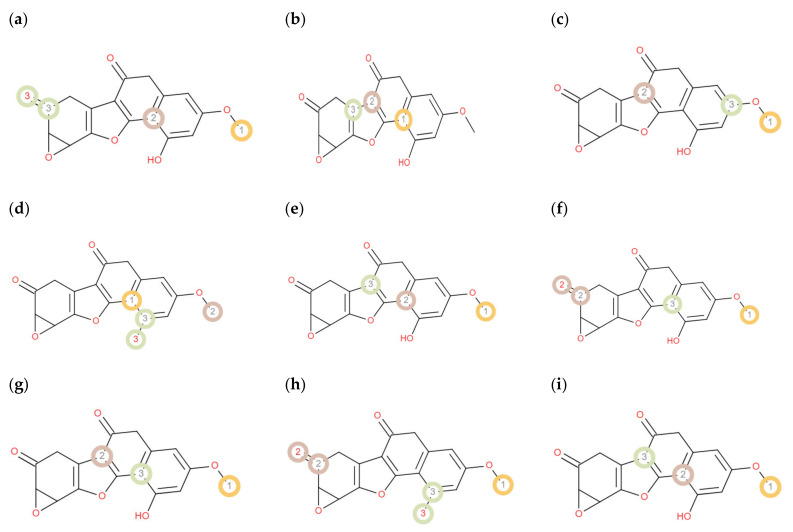
Prediction of DL site of metabolism by RS-Predictor at: (**a**) CYP1A2, (**b**) CYP2A6, (**c**) CYP2B6, (**d**) CYP2C8, (**e**) CYP2C9, (**f**) CYP2C19, (**g**) CYP2D6, (**h**) CYP2E1, (**i**) CYP3A4. Color code: orange: highly labile, grey: moderately labile, and light green: fairly labile.

During the preclinical development of the drugs, the prediction of the metabolism of any drug candidate at the various site cytochrome 450 is vital because this can help to avoid the withdrawal of the tested molecules in later stages and also lowers the risks connected with biotransformation [28]. Therefore, the metabolic liability of DL was also prophesied because its metabolites and their reactivity are still unidentified. The assessment of DL metabolism at different site of CYP enzymes is also important because in this way we can avoid the pharmacokinetic interactions. In this study, two diverse programs pkCSM [29] and RS-Predictor [30] were used for the estimation of DL metabolism. The pkCSM package was used to define the metabolism probability of DL at different cytochrome P450 enzymes (3A4, 1A2, 2C19, 2C9, and 2D6). The results showed that DL is metabolized only by CYP 1A2 and 2C9 (Table 10), which exhibited a good indication of prominent anticancer agent. The RS-Predictor was used to recognize SOM (site of metabolism) and its’ possibilities for CYP enzyme (1A2, 2A6, 2B6, 2C8, 2C9, 2C19, 2D6, 2E1 and 3A4) (Figure 4). The assessment exhibited that CH3 group and a carbon atom of naphthalene ring are extremely probable to be SOM’s. However, other parts of the DL were recognized as not so likely to be SOM’s. Organic cation transporter 2 (OCT2) is a renal reabsorption transporter which plays an important role in renal clearance of drug-like substances. Therefore, the valuation of OCT2 for a drug candidate delivers valuable evidence about its clearance [28]. The study illustrated that DL is distinct from a substrate for OCT2. Due to its lower transportation tendency by OCT2, it can be implicit that DL will strongly bind with plasma proteins and exhibit long-lasting pharmacological effect.

## 3. Materials and Methods

DEN purchased from the Sigma Aldrich Chemical Company, St. Louis, MI, USA. All of the other chemical, reagent and kits were of analytical reagent grade and purchased from the approved vendor.

### 3.1. Experimental Animals

Adult male Wistar (Chakraborty Enterprise, Kolkata, India, Swiss albino strain) (weight range 150–200 gm) were acquired from the registered vendor for the experiment. The animals were familiarized to metabolic cage under standard experimental laboratory condition (12 h light/dark cycle; 22 ± 2 °C; relative humidity 30–50%) with standard pellet diet and water as ad libitum. All of the the animals were acclimatized for 15 days prior to experiment. All of the the experiments were conducted according to Institutional Animal Ethical Committee of Sam Higginbottom University of Agriculture, Technology and Sciences (approval letter no: 1813/GO/RE/S/15/CPCSEA/20210728/02) for the Purpose of Control and Supervision of Experiments on Animals, Government of India.

#### 3.1.1. Induction of Hepatocellular Carcinoma

Diethyl nitrosamine (DEN) was prepared in a phosphate buffer solution and a single dose of intraperitoneal injection were administered to animals. [31]. Liver cancer was confirmed by the estimation of the alpha fetoprotein (AFP) after the 7 days. Experimental design

After successfully induction the HCC, the rats were divided into following groups:

Group 1: Normal control received vehicle
Group 2: Normal control + DL (10 mg/kg)
Group 3: DEN control: Administered vehicle only
Group 4: DEN control + DL (2.5 mg/kg)
Group 5: DEN control + DL (5 mg/kg)
Group 6: DEN control + DL (10 mg/kg)

All of the groups received the drugs and vehicle once in a day for 22 weeks. Water/food intake and behavioral changes were regularly monitored.

#### 3.1.2. Estimation of the Biochemical Parameters

The blood samples of the experimental rats were directly collected from the retro-orbital plexus under diethyl ether. The blood samples were kept at room temperature for 30 min. Further, the blood samples were centrifuged at 3000 rpm for 15 min, separated the serum, and stored in between 2–4 °C for the further use. The liver parameter and hematological parameters such as serum glutamate pyruvate transaminase (SGPT), serum glutamate oxaloacetate transaminase (SGOT), alpha fetoprotein (AFP), gamma-glutamyltranspeptidase, serum alkaline phosphates (SAP), nitric acid (NO), white/red blood cells (WBC/RBC), erythrocytes sedimentation rate (ESR), hemoglobin (HB), mean corpuscular volume (MCV), packed cell volume (PCV), mean corpuscular hemoglobin concentration (MCHC), and mean corpuscular hemoglobin (MCH) were analyzed according to the standard kits [32,33,34,35].

#### 3.1.3. Assessment of Inflammatory Mediators and Proinflammatory Cytokines

The estimation of inflammatory mediator (NF-kB) and proinflammatory cytokines (IL-1β, TNF-α, and IL-6) was conducted using enzyme-linked immune sorbent Assay (ELISA) standard kits. The procedure and instructions were provided by the manufacturer.

#### 3.1.4. Histopathological Studies

The liver tissue of the experimental animals was subjected to histopathological examination. Therefore, the hepatic tissue of rats was fixed in 40% formalin and embedded in paraffin wax, stained with hematoxylin and eosin for the processing.

### 3.2. In Silico Absorption, Distribution, Metabolic Liability Prediction, and Excretion Studies

The calculation of *insilico* physicochemical properties such as logP, GI absorption, Lipinski violations, H donor/acceptor, polar surface area (TPSA), blood brain permeability probability, metabolism at the site of CYP450, and renal OCT2 substrate clearance were measured using the three online platforms www.chemosophia.com (accessed on 15 June 2021) [36], Swissadme.ch (accessed on 15 June 2021) [37], and PkCsM [29]. However, the metabolism liability of DL at various cytochrome P450 enzymes was calculated using another web server RS Predictor [32].

### 3.3. Statically Analysis

The data are expressed as mean ± SEM for each group. Statistical analysis was performed using GraphPad Prism version 5.0. One-way analysis of variance (ANOVA) followed by Dunnett’s *t*-test.

## 4. Conclusions

The study illustrated that DL may suppress the formation of free radicals and inflammatory mediators such as lipid peroxidation, IL-1β, TNF-α and IL-6 in DEN-induced hepato-carcinogenesis (Wistar strain) by improving the level of endogenous antioxidant through scavenging the free radicals and decline the secretion of inflammatory mediators form the Kupffer cells. Therefore, the present research showed the oppressive effect of DL in the progression of tumors in DEN-induced HCC, which may comprise the antioxidant and anti-inflammatory mechanism. The detailed molecular mechanism of action of debelalactone against DEN-induced hepato-carcinogenesis is under way. Additionally, insilico studies established that DL has a great affinity to interact with the DNA antimetabolites receptor. The introduction of an epoxide function into a structural backbone is still one of the potential modifications being implemented in drug design despite of the long-standing controversy that it has a high electrophilic nature. In anticancer drug discovery, epoxide compounds (for example, arglabin, and germacrone) widly incouraged because these compounds inactivate DNA synthesis by formation of interstrand cross-links. Therefore, epoxides can also have significant interest in medicinal chemistry, affording molecules with undeniable therapeutic value. Bearing this in mind, it is our strong opinion, that epoxides such as DL can be a suitable option anticancer agents and deserve a better exploration.

## Figures and Tables

**Table 1:** Effect of DL on body and liver weight. This section may be divided by subheadings. It should provide a concise and precise description of the experimental results, their interpretation, as well as the experimental conclusions that can be drawn.

Groups	Initial Body Weight (gm)	Final Body Weight (gm)	Liver Weight (gm)
Normal Control	105.9 ± 0.66	324.6 ± 0.86	14.3 ± 0.51
Normal Control + DL (10 mg/kg)	117.3 ± 1.20	311.5 ± 1.96	15.06 ± 0.21
DEN Control	129.9 ± 0.96 *	295.3 ± 2.33	18.63 ± 0.50
DEN Control + DL (2.5 mg/kg)	124.7 ± 1.27 *	306.8 ± 1.65	17.03 ± 0.12 *
DEN Control + DL (5 mg/kg)	124.8 ± 1.8 ^ns^	319.9 ± 1.68 **	15.06 ± 0.14 ***
DEN Control + DL (10 mg/kg)	108.7 ± 1.65 ^ns^	342.1 ± 2.47 ***	14.5 ± 0.15 ***

All values represent mean ± SEM * *p* < 0.05; ** *p* < 0.01; *** *p* < 0.001, ns = non-significant; ANOVA, followed by Dunnett’s multiple comparison test.

**Table 2:** Effect of DL on non-liver parameters.

Parameters	Groups					
	Normal Control	Normal Control + DL (10 mg/kg)	DEN Control	DEN Control + DL (2.5 mg/kg)	DEN Control + DL (5 mg/kg)	DEN Control + DL (10 mg/kg)
NO (µM/L)	31.7 ± 0.77	31.2 ± 0.49	61.9 ± 0.96	55.7 ± 0.54 ^ns^	45.2 ± 0.35 **	35.4 ± 0.57 ***
AST (U/L)	71.5 ± 0.65	71.2 ± 0.78	191.5 ± 0.72	153.6 ± 0.67 *	125.7 ± 0.65 ***	85.5 ± 0.65 ***
ALT (U/L)	41.8 ± 0.66	41.5 ± 0.52	104.6 ± 0.55	90.5 ± 0.46 **	64.3 ± 0.38 ***	44.9 ± 0.74 ***
ALP (U/L)	74.7 ± 0.64	75.13 ± 0.42	190.8 ± 0.7	160.2 ± 1.09 *	126.9 ± 0.80 **	85.7 ± 0.84 ***

All values represent mean ± SEM * *p* < 0.05; ** *p* < 0.01; *** *p* < 0.001, ^ns^ = non-significant; ANOVA, followed by Dunnett’s multiple comparison test.

**Table 3:** Effect of DL on liver parameters.

Parameters	Groups					
	Normal Control	Normal Control + DL (10 mg/kg)	DEN Control	DEN Control + DL (2.5 mg/kg)	DEN Control + DL (5 mg/kg)	DEN Control + DL (10 mg/kg)
Total protein (mg/dL)	8.4 ± 0.17	8.4 ± 0.24	3.9 ± 0.14	5.3 ± 0.14 *	6.1 ± 0.14 **	7.5 ± 0.12 ***
Alpha fetoprotein (mg/dL)	21.2 ± 0.72	20.7 ± 0.28	308 ± 1.01	125.4 ± 0.81 ***	70.8 ± 0.89 ***	28.73 ± 0.73 ***
Albumin (mg/dL)	4.2 ± 0.15	4.43 ± 0.24	1.7 ± 0.05	1.9 ± 0.19 ^ns^	2.83 ± 0.23 *	3.6 ± 0.20 ***
Blood urea nitrogen (BUN) (mg/dL)	18.8 ± 0.26	19.1 ± 0.25	34.4 ± 0.54	29.4 ± 0.51 *	26.4 ± 0.67 **	20.8 ± 0.89 ***
Total Bilirubin (mg/dL)	11.1 ± 0.12	12.13 ± 0.18	54.6 ± 0.49	43.9 ± 0.69 *	31.5 ± 0.59 ***	15.7 ± 0.50 ***
Direct Bilirubin (mg/dL)	6.3 ± 0.46	6.1 ± 0.53	18.1 ± 0.33	17.1 ± 0.38 *	13.8 ± 0.23 **	9.2 ± 0.40 ***

All values represent mean ± SEM * *p* < 0.05; ** *p* < 0.01; *** *p* < 0.001, ^ns^ = non-significant; ANOVA, followed by Dunnett’s multiple comparison test.

**Table 4:** Effect of DL on hematological parameters.

Parameters	Groups					
	Normal Control	Normal Control + DL (10 mg/kg)	DEN Control	DEN Control + DL (2.5 mg/kg)	DEN Control + DL (5 mg/kg)	DEN Control + DL (10 mg/kg)
WBC (10^3^/mm^3^)	9.1 ± 0.41	8.9 ± 0.16	14.5 ± 0.49	14.3 ± 0.27 ^ns^	12.5 ± 0.49 *	10.7 ± 0.35 ***
RBC (10^6^/mm^3^)	6.2 ± 0.38	5.9 ± 0.27	1.8 ± 0.38	2.9 ± 0.38 *	3.6 ± 0.44 **	4.7 ± 0.23 ***
Platelet count (10^3^/mm^3^)	920.3 ± 1.19	920.6 ± 0.57	1152.7 ± 1.01	1082.2 ± 1.0 *	1031.5 ± 1.01 **	960.9 ± 0.92 ***
Hb (gm/dL)	14.5 ± 0.49	14.5 ± 0.43	7.3 ± 0.52	8.6 ± 0.34 *	9.7 ± 0.33 **	12.5 ± 0.47 ***
ESR (mm/hr)	8.6 ± 0.18	9.03 ± 0.24	13.4 ± 0.21	12.3 ± 0.42 *	11.5 ± 0.56 **	9.5 ± 0.53 ***
PCV (%)	42.7 ± 0.69	40.2 ± 0.69	31.1 ± 0.55	33.4 ± 0.56 *	35.5 ± 0.76 **	41.4 ± 0.64 ***
MCV (fl)	55.4 ± 0.51	55.7 ± 0.46	60.3 ± 0.61	58.5 ± 0.66 ^ns^	57.1 ± 0.01 *	56.5 ± 0.34 ***
MCH (pg)	19.5 ± 0.64	19.2 ± 0.56	15.6 ± 0.56	15.8 ± 0.61 ^ns^	16.9 ± 0.56 *	19.5 ± 0.64 ***

All values represent mean ± SEM * *p* < 0.05; ** *p* < 0.01; *** *p* < 0.001, ^ns^ = nonsignificant, WBC = White blood cell, RBC = Red blood cell, Hb = Hemoglobin, ESR = Erthrocyte sedimentation rate, PCV = Packed cell volume, MCV = Mean corpuscular volume, MCH = Mean corpuscular hemoglobin; ANOVA, followed by Dunnett’s multiple comparison test.

**Table 5:** Effect of DL on endogenous antioxidant markers.

Parameters	Groups					
	Normal Control	Normal Control + DL (10 mg/kg)	DEN Control	DEN Control + DL (2.5 mg/kg)	DEN Control + DL (5 mg/kg)	DEN Control + DL (10 mg/kg)
LPO (µM/mg protein)	6.5 ± 0.38	6.9 ± 0.08	14.6 ± 0.41	13.9 ± 0.52 **	11.9 ± 0.27 ***	7.6 ± 0.35 ***
CAT (nmol/min/mL)	0.87 ± 0.05	0.85 ± 0.03	0.32 ± 0.02	0.43 ± 0.02 *	0.58 ± 0.01 **	0.84 ± 0.01 ***
SOD (U/mL)	1.8 ± 0.02	1.7 ± 0.02	0.88 ± 0.02	1.5 ± 0.23 *	1.37 ± 0.01 **	1.7 ± 0.02 ***
GPx (µmol)	8.7 ± 0.15	8.6 ± 0.06	2.6 ± 0.17	4.6 ± 0.15 **	5.7 ± 0.19 ***	7.5 ± 0.22 ***
GST (U/min/mg protein)	0.23 ± 0.01	0.23 ± 0.01	0.05 ± 0.01	0.08 ± 0.01 *	0.15± 0.01 **	0.19 ± 0.01 ***

All values represent mean ± SEM * *p* < 0.05; ** *p* < 0.01; *** *p* < 0.001, LPO = Lipid peroxidation, CAT = Catalase, SOD = Superoxide dismutase, GPx = Glutathione peroxidase, GST = glutathione transferase; ANOVA, followed by Dunnett’s multiple comparison test.

**Table 6:** Effect of DL on proinflammatory markers.

Parameters	Groups					
	Normal Control	Normal Control + DL (10 mg/kg)	DEN Control	DEN Control + DL (2.5 mg/kg)	DEN Control + DL (5 mg/kg)	DEN Control + DL (10 mg/kg)
TNF-α (pg/mL)	42.9 ± 0.93	41.2 ± 0.85	149.4 ± 1.22	131.4 ± 1.09 **	96.7 ± 0.81 ***	67.7 ± 1.13 ***
IL-1β (pg/mL)	22.9 ± 0.75	21.9 ± 0.33	94.9 ± 1.41	69.8 ± 0.80 **	47.2 ± 0.82 ***	31.2 ± 0.79 ***
IL-6 (pg/mL)	92 ± 0.92	90.4 ± 0.52	230.8 ± 1.51	191.9 ± 1.04 *	152.1 ± 1.22 **	111.1 ± 1.12 ***
NF-kB (ng/mL)	121.2 ± 0.84	120.4 ± 0.54	197.6 ± 1.22	175.6 ± 1.11	151.2 ± 1.03	132.7 ± 1.30

All values represent mean ± SEM * *p* < 0.05; ** *p* < 0.01; *** *p* < 0.001, TNF-α = Tumor necrosis factor–α, IL-1β = interleukin-1β, IL-6 = interleukin-6; ANOVA, followed by Dunnett’s multiple comparison test.

**Table 7:** Effect of debelalactone on DEN-induced hepatocarcinogenesis in rats.

Histopathology	Groups					
	Normal Control	Normal Control + DL (10 mg/kg)	DEN Control	DEN Control + DL (2.5 mg/kg)	DEN Control + DL (5 mg/kg)	DEN Control + DL (10 mg/kg)
Necrosis	-	-	+	+	+	-
Apoptosis	-	-	+	+	+	-
Hydropic degeneration	-	-	+	+	+	-
Pseudo-nucleoli	-	-	+	+	-	-
disorganized hepatic parenchyma	-	-	+	+	-	-
Bile cysts	-	-	+	+	+	-
Peliosis hepatis	-	-	+	+	-	-
Hyperplastic foci	-	-	+	+	+	-
Diffuse dysplasia	-	-	+	+	+	-
Hepatocelluar adenoma	-	-	+	+	-	-
cell necrosis	-	-	+	+	+	-
small dark cytoplasm	-	-	+	+	-	-
Altered basophilic	-	-	+	+	+	-
Macro lipid droplets	-	-	+	+	-	-
Enlargement of karyomegali	-	-	+	+	+	+
HSCs focal proliferation	-	-	+	+	-	-

**Table 8:** Predicted probability of anticancer activity of DL for different mechanisms of action (alkylating action (Alk), antimitotics (AMi), inhibitors of topoisomerase 1 (TI-1), topoisomerase 2 (TI-2), dihydrofolate reductase (DHFR), cyclin-dependent kinase (CDK4), epidermal growth factor receptor (EGFR), cDNA—DNA—antimetabolites of cancer cells.

Compound	Alk	AMi	TI-1	DHFR	cDNA	TI-2	CDK4	EGFR
DL (stereoisomer 1)	0.4113	0.0485	0.3108	0.3061	0.8499	0.0227	0.6265	0.9340
DL (stereoisomer 2)	0.6381	0.0025	0.2584	0.2128	0.8679	0.0182	0.5540	0.9159
1ims (ligand)					0.8787			

**Table 9:** Characteristics of the ligand-cDNA complementarity using (CF1): squared correlation coefficient—R2, standard error of estimate (Sigma), maximum of CF1 (MaxCF1).

Compound	R2	Sigma	Npoints	MaxCF1
DL1	0.882	0.32	11839	−4.068
DL2	0.889	0.28	11126	−4.551
1d37	0.860	0.32	16115	−4.451
1ims	0.895	0.30	17358	−4.410

**Table 10:** DL physiochemical properties (ADME).

Name	log*P*	GI abs	Lip vio	H acc	H don	TPSA	BBB per	Metabolism at CYP450					Renal OCT2 Substrate
								3A4	1A2	2C19	2C9	2D6	
DL	1.96	H	N	6	1	89.27	N	N	Y	N	Y	N	N

Note: logP is the logarithm of the octanol–water partition coefficient; GI abs: gastrointestinal absorption; Lip vio: total number of Lipinski’s rule of five violations; H acc: hydrogen acceptor; H don: hydrogen donor; TPSA: total polar surface area; Columns CYP3A4, CYP1A2, CYP2C19, CYP2C9, and CYP2D6 show metabolism on different site of cytochrome P450; Renal OCT2 substrate: Organic cation transporter 2.

## Data Availability

The data presented in this study are available in this article.

## Abbreviations

DEN = Diethylnitrosamine, HCC = Hepatocellular Carcinoma, AFP = Alpha fetoprotein, SGOT = Serum glutamate oxaloacetate transaminase, SGPT = Serum glutamate pyruvate transaminase, GGTP = Gamma-glutamyltranspeptidase, ALP = Serum alkaline phosphatase, ALT = Alanine aminotransferase, AST = Aspartate aminotransferase, NO = Nitric oxide, RBC = Red blood cells, WBC = White blood cells, Hb = Hemoglobin, ESR = Erythrocytes sedimentation rate, PCV = Packed cell volume, MCV = Mean corpuscular volume, MCH = Mean corpuscular hemoglobin, TP = Total protein, AB = Albumin, BUN = Blood urea nitrogen, TB = Total bilirubin, DB = Direct bilirubin, LDH = Lactate dehydrogenase, K = Potassium, Na = Sodium, LPO = Lipid peroxidation, CAT = Catalase, SOD = Superoxide dismutase, GPx = Glutathione peroxidase, GST = glutathione transferase, VEGF = Vascular endothelial growth factor, TNF-α = Tumor necrosis factor α, IL-1β = interleukin-1β, IL-6 = interleukin-6.
